# Establishment of an Internet-Based Epidemiological Survey Data Collection Customized System Model

**DOI:** 10.3389/fpubh.2021.761031

**Published:** 2021-10-15

**Authors:** Xusheng Wang

**Affiliations:** Faculty of Printing, Packaging Engineering and Digital Media Technology, Xi'an University of Technology, Xi'an, China

**Keywords:** internet age, epidemiological study, data collection, system model, collection customized system model

## Abstract

Epidemiology occupies a very important position in preventive medicine. Its essence is to summarize the etiology and epidemic laws by studying the distribution and possible effects of diseases, so as to promote the formation of scientific epidemic prevention measures. The purpose of this article is to help relevant personnel complete the collection, induction, and analysis of epidemiological survey data by establishing a data collection system model to improve work efficiency. This article focuses on the new coronavirus pneumonia (COVID-19), investigates the development status of epidemiological survey data collection, and analyzes the problems in the current business process, and on this basis, develops a dedicated epidemiological survey System model for data collection. From the experimental data, the optimized correction evaluation index has been increased from 8.384 to 9.067. It can be seen that the combination of data mining algorithms and backpropagation algorithms can better improve the system's ability to process information. Professional information disclosure platforms can have a good positive impression on the prevention and treatment of epidemics. The Internet-based epidemiological survey customized system model established in this article is to integrate various epidemiological data so that people can correctly understand the spread of epidemics and promote the development of preventive medicine.

## Introduction

For a long time, epidemics have affected people's health and property safety, and they are extremely destructive and harmful. In recent years, various epidemics have broken out globally, such as measles, malaria, tuberculosis and so on. In addition, emerging epidemic problems are also increasing year by year, such as the new coronavirus pneumonia (COVID-19) that has gradually swept the world from the end of 2019. How to contain the outbreak of epidemics and alleviate the spread of diseases has become an urgent problem that must be faced by today's society. Considering the particularity of the epidemiological pathology, large-scale experiments cannot be carried out. Therefore, we can only simulate the spread of the disease through theory, predict the spread of the disease, and formulate reasonable prevention and control measures. In order to achieve this goal, it is very necessary to establish a special epidemiological survey data collection system model.

Data research on epidemiology has a history of many years abroad. Staa used a large drug epidemiological database to study oral corticosteroids and the risk of fractures. In order to assess the sensitivity of the relationship between oral corticosteroid use and fracture risk, he and his team collected a large number of patient data from the general medical research database as a strong basis for the study. Compared with a comprehensive general medicine database, if data is obtained from a specialized epidemiological drug database, the conviction of experimental results can be further enhanced (1). Gaudart studied the performance comparison between multilayer perceptron and linear regression in epidemiological data processing. The development of computer and Internet technology provides more powerful technical support for epidemiological data research, and neural networks are increasingly used as statistical models. Taking into account deviations such as normality, isomorphism, and error independence, Gaudart designed five designs and conducted a data simulation for each design. Judging from the experimental data, the prediction results have good stability, but more sample data are needed as evidence (2).

In recent years, domestic research on epidemiological survey data in the Internet age has also achieved fruitful results. Lin C F conducted research on the epidemiology of dyslipidemia in Asia-Pacific countries. In order to compare the epidemiology of dyslipidemia in the elderly and the general population in the Asia-Pacific region, he and his team collected differences in the prevalence of dyslipidemia in the elderly in China, Japan, South Korea, Malaysia and other countries. From the experimental data, the prevalence of high total cholesterol (TC) and high-low-density lipoprotein cholesterol (LDL-C) in most countries is higher than that of the general population. As the research objects involve various countries in the Asia-Pacific region, there are many difficulties in collecting epidemiological data. If a comprehensive data system model can be established based on the Internet, it will undoubtedly be of great help to improve research efficiency (3).

Based on the Internet and computer technology, this paper conducts in-depth research on the establishment of a customized system model for epidemiological survey data collection. The research is mainly carried out from the following aspects: First, this article introduces the related technologies and methods involved in the establishment of the system model, including Internet-based data collection systems, epidemiological data models, data mining algorithms, and anti-corruption systems. To the propagation algorithm. Secondly, after analyzing the problems and deficiencies in the original survey business, this article combines Internet technology with its key business processes, and uses the Architecture Modeling Method (IDEF) to complete the establishment of the epidemiological survey data customization system model. Finally, this article gives a detailed description of the collection and analysis of system data and the impact of the COVID-19 epidemic on global information management.

## Internet-Based Epidemiological Survey Data Collection System Technology

### Internet-Based Data Acquisition System

To use a computer to process and display information in the real world, you must first connect the computer with the real world. This needs to convert various signals in the real world into signals that can be identified and stored by the computer. This process is data acquisition (4, 5). Data acquisition technology is a comprehensive technology based on front-end analog signal processing, analog signal digitization, digital signal processing and computer control technology and other high-tech, which has been widely used in many fields (6). The development of digital technology promotes the progress of these fields to a certain extent. On the other hand, it puts forward higher requirements for data acquisition system. A large data acquisition system consists of the following parts: data measurement, data acquisition, data transmission, data storage, data processing, analysis and display (7).

In today's networked era, the development and application of computer network communications represented by the Internet has achieved unprecedented breakthroughs and successes. Networked measurement, acquisition and control technologies are developing with the development of network technology (8). The advantages of networked and distributed data collection are reflected in the expansion of the collection range, enhanced processing capabilities, more convenient information retrieval, and the ability to adapt to the changing needs of occasions (9). With these excellent performances, networked measurement and control have become an inevitable trend in the development of data acquisition technology. The rapid development of sensor technology, computer technology and network technology and the resulting measurement requirements have become an inexhaustible driving force for the development of data acquisition technology, and proposed newer and higher requirements for measurement methods and measurable data acquisition technology. The type and scope should be continuously expanded and updated, the accuracy should be improved, the reliability should be enhanced, and be able to adapt to the needs of various experimental environments (10, 11).

### Data Model Based on Epidemiology

A lot of information will be involved in the epidemiological survey data collection process. For this reason, the data collection method and the authenticity of the information should be scientifically analyzed in the process of its realization (12). At the same time, due to the rapid development of science and technology and a large number of applications in practice, many advanced data acquisition and analysis technologies have emerged. For medical and health organizations, in order to improve work efficiency and calmly respond to changes in real-time information, a deep understanding and skilled use of data mining (DM) models are particularly important (13, 14).

Since the outbreak of the novel coronavirus pneumonia (COVID-19), my country has adopted various measures to combat the epidemic. Among them, the real-time and accurate release of information on the epidemic by local governments has played a great role in stabilizing people's sentiment and controlling the epidemic. The openness and transparency of my country has made China praised by the world (15). From a professional point of view, the survey data on epidemics can allow the government and professional institutions to have an accurate grasp of the real-time spread and spread of the disease, so as to better make the corresponding counterparts; from a social point of view, the official The given professional information data can also prevent the masses from falling into panic and increase the masses' sense of trust in the government (16).

### Data Mining Algorithm

With the rapid development of database technology, database management system has been more and more widely used. People have accumulated more and more data, and there are many important information hidden behind the surge of data. People hope to analyze it at a higher level in order to make better use of these data (17). The current database system can efficiently implement data entry, query, statistics and other functions, but it cannot find the relationships and rules in the data, and cannot predict the future development trend based on the existing data (18). The lack of the means of mining the hidden knowledge behind the data has led to the phenomenon of “explosive data but poor knowledge”. While a large amount of information brings convenience to people, it also brings a lot of problems: too much information, difficult to digest; difficult to distinguish between true and false; difficult to guarantee security; different forms, difficult to unified processing, etc. In the face of these challenges, data mining technology emerged and showed strong vitality (19, 20).

Association rules and cluster analysis are the two most common data mining methods. Apriori algorithm is a typical algorithm in association rules, and K-means algorithm is the core algorithm in cluster analysis (21, 22). This article will briefly describe the flow of the K-means algorithm. First, suppose the original data set is:


(1) $$\begin{array}{l} X=\left\{x1,x2,x3,\cdots \;,xn\right\} \end{array}$$

After giving the number of classes *K*, randomly selectV points from the data set as the starting cluster, and use the Euclidean distance formula as the distance measurement standard to measure, and calculate the distance between each point and the *K* cluster centers., The new cluster center can be obtained:


(2) $$\begin{array}{l} Cj=\frac{1}{nj}\underset{xm\in cj}{\sum }xm,1\leq j\leq K \end{array}$$

Set the objective function as:


(3) $$\begin{array}{l} J={\overset{K}{\underset{j=1}{\sum }}\underset{xi\in Cj}{\sum }|xi-cj|}^{2} \end{array}$$

**Figure 1:**
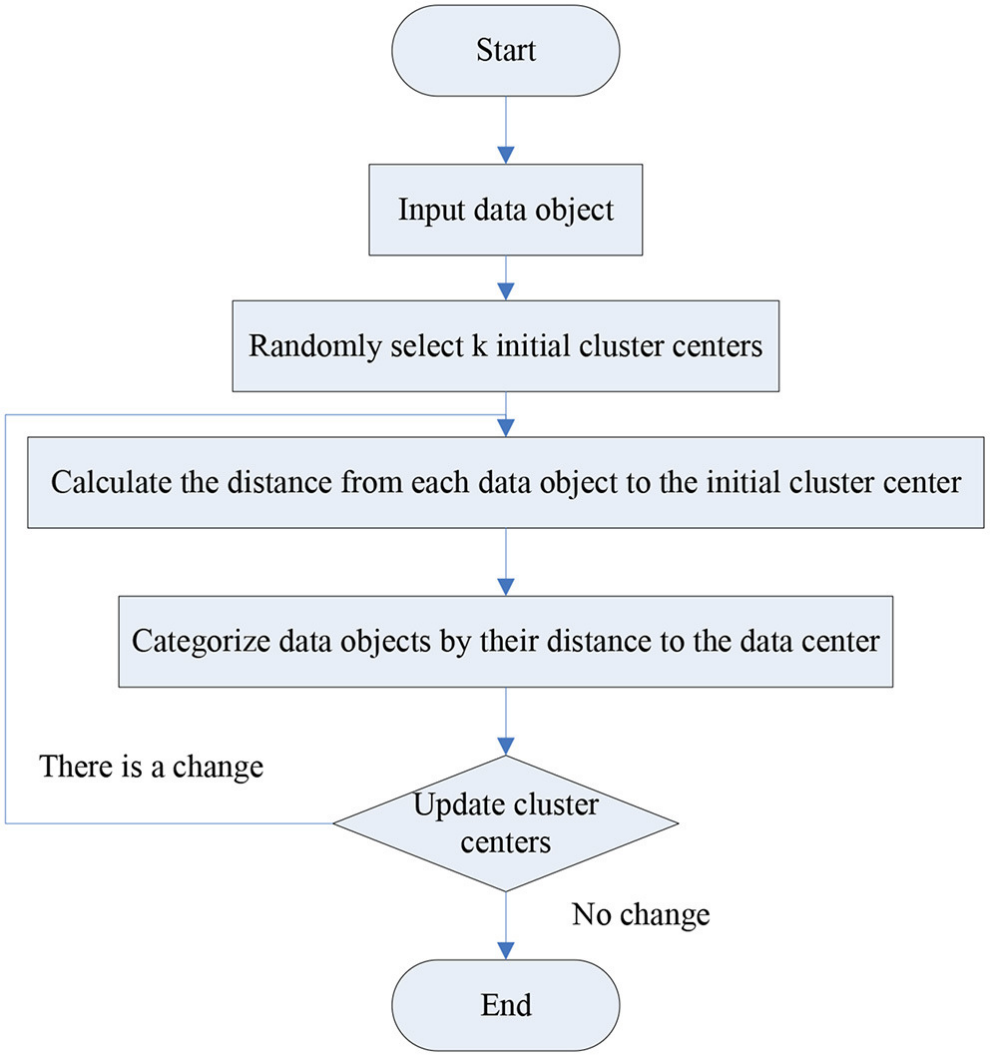
Flow chart of K-means algorithm.

The flow of the K-means algorithm is shown in Figure 1 below.

### Backpropagation Algorithm

The multi-layer feedforward network learning algorithm is more complicated, because the hidden layer in the middle is not directly connected to the outside world, and its error cannot be directly calculated (23). In order to solve this problem, people found back propagation (BP) algorithm in the research process. Its main idea is to propagate the error of output layer layer layer by layer from back to front to calculate the error of hidden layer indirectly. In the back-propagation algorithm, in order to speed up the convergence speed, the gradient method can be used to modify the weight (24). Considering that the output function must be differentiable, the Sigmoid function can be used:


(4) $$\begin{array}{l} f\left(x\right)=\frac{1}{1+\exp\left(-x\right)} \end{array}$$

Given that the input vector is *X* = (*x*1, *x*2, ⋯  , *xn*)^*T*^, the hidden layer output vector is *Y* = (*y*1, *y*2, ⋯  , *yh*)^*T*^, the output layer vector *O* = (*o*1, *o*2, ⋯  , *om*)^*T*^, and the expected output vector is *d* = (*d*1, *d*2, ⋯  , *dm*)^*T*^, the input and output of the *k*th neuron in the output layer are:


(5) $$\begin{array}{l} netk=\overset{h}{\underset{j=1}{\sum }}wjkyj\;k=1,2,\cdots \;,m \end{array}$$

(6) $$\begin{array}{l} ok=f\left(netk\right)\;k=1,2,\cdots \;,m \end{array}$$

The input and output of the *j* th neuron in the hidden layer are:


(7) $$\begin{array}{l} netj=\overset{n}{\underset{i=1}{\sum }}vjkxi\;j=1,2,\cdots \;,h \end{array}$$

(8) $$\begin{array}{l} yj=f\left(netj\right)\;j=1,2,\cdots \;,h \end{array}$$

Generally speaking, the actual output of the network is not equal to the expected output, that is, there is an error *E*, and its definition satisfies:


(9) $$\begin{array}{l} E=\frac{1}{2}{\left(d-o\right)}^{2}=\frac{1}{2}{\overset{m}{\underset{k=1}{\sum }}\left(dk-ok\right)}^{2} \end{array}$$

The principle of adjusting network weights is to continuously reduce the error, so the adjustment of the weights should be proportional to the negative gradient of the error, namely:


(10) $$\begin{array}{l} \Delta wjk=-\eta \frac{\partial E}{\partial wjk}=-\eta \frac{\partial E}{\partial netk}•\frac{\partial netk}{\partial wjk}\;j=1,2,\cdots \;,h\; \end{array}$$

(11) $$\begin{array}{l} \Delta vij=-\eta \frac{\partial E}{\partial vij}=-\eta \frac{\partial E}{\partial netj}•\frac{\partial netj}{\partial vij}\;i=1,2,\cdots \;,h \end{array}$$

At the same time, the error function can be further written as:


(12) $$\begin{array}{l} E=\frac{1}{2}{\left(d-o\right)}^{2}=\frac{1}{2}{\overset{m}{\underset{k=1}{\sum }}\left[dk-f\left(\overset{h}{\underset{j=1}{\sum }}wjkyj\right)\right]}^{2} \end{array}$$

TTherefore, the calculation formula of the weight adjustment from the hidden layer to the output layer is:


(13) $$\begin{array}{l} \Delta Wjk=\eta \left(dk-ok\right)f^{\prime }\left(netk\right)yj \end{array}$$

The calculation formula for weight adjustment from the input layer to the hidden layer is:


(14) $$\begin{array}{l} \Delta Wij=\eta \overset{m}{\underset{k=1}{\sum }}\left[\left(dk-ok\right)f^{\prime }\left(netk\right)wjk\right]f^{\prime }\left(netj\right)xi \end{array}$$

If the Sigmoid function is selected for the output of the hidden layer and the output layer, consider:


(15) $$\begin{array}{l} f^{\prime }\left(x\right)=f\left(x\right)\left[1-f\left(x\right)\right] \end{array}$$

The variants of formulas (13) and (14) are:


(16) $$\begin{array}{l} \Delta Wjk=\eta \left(dk-ok\right)ok\left(1-ok\right)yj \end{array}$$

(17) $$\begin{array}{l} \Delta Wij=\eta \overset{m}{\underset{k=1}{\sum }}\left[\left(dk-ok\right)ok\left(1-ok\right)wjk\right]yj\left(1-yj\right)xi \end{array}$$

In practical applications, training samples should be input when learning. Each time all training samples are input is called a training cycle. Learning should be performed cycle by cycle until the objective function is generally the error function reaches the minimum or less than a given value (25, 26).

## Experiment on the Construction of an Internet-Based Epidemiological Data Collection System

### Experimental Background

To realize the construction of an effective epidemiological survey data collection system model, the cooperation between multiple links is required, among which the most basic is the collection of information and data. In the past, most of the data collection methods were implemented by staff through on-site surveys or telephone surveys. In the context of the Internet, online questionnaire surveys have also become an important channel for epidemiological data collection. Because epidemics often have the characteristics of short cycles and fast timeliness, in terms of timeliness, network systems are obviously more efficient than manual investigations. With this in mind, the design of questionnaires and data entry have become a very critical part of building a data collection system. In this era of big data, how to select valuable information from massive amounts of data to provide an effective basis for scientific decision-making is an important research direction for network system platform management and development.

### Experimental System Development

**Figure 2:**
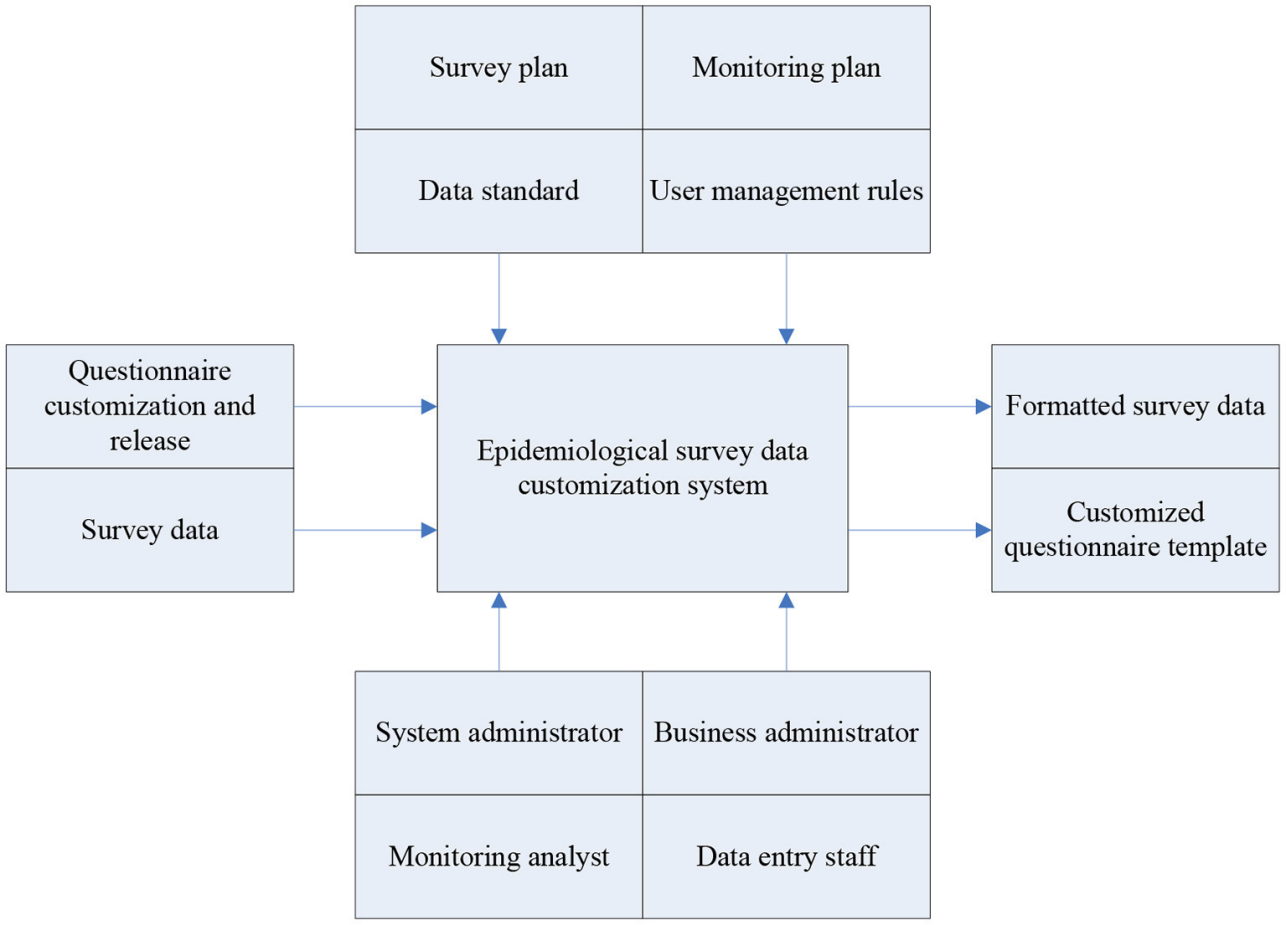
Epidemiological survey data system model.

After analyzing the problems and deficiencies in the original survey business, this paper combines Internet technology with its key business processes, and uses the Architecture Modeling Method (IDEF) to complete the establishment of a customized system model for epidemiological survey data. IDEF is a commonly used structural analysis method. In the research process of this article, the main goal is to build a system that can effectively realize data input, control and mechanism. Input is the element transformed by the function in the system, the control will determine the execution of the function, and the mechanism represents the resources needed to perform the activity. Figure 2 shows the epidemiological survey data system model.

It can be seen from Figure 2 that when setting the system model, this article first considers the design of the survey plan and the testing plan. After completing the construction of the general plan structure in combination with the system goals, the formulation of the questionnaire, the collection of survey data and the data standard A definition is given. Finally, this article also formulated relevant management rules for system users. The system administrator has the authority to manage all members, while data entry personnel and monitoring and analysis personnel are responsible for ensuring the normal operation of the system.

### System Model of Experimental Components

When designing the epidemiological data collection model, this article hopes to give the system professionalism and a certain versatility at the same time, so as to avoid restarting the system every time a new epidemic occurs, and adding unnecessary workload. Considering that the content of epidemiological investigation involves many professional domain knowledge and diverse information, this article will realize the establishment of business model and data model based on components.

The essence of the component-based experimental model is to combine the analysis of business data to encapsulate the survey content and effectively achieve the effect of reuse. For example, when designing a questionnaire, in addition to a special database for new epidemics, many basic information components can be used repeatedly. For example, the user's name, gender, and ID number in the survey object information component; the time, location, and event level in the event component; the person who filled the form and the unit in the management information component, etc.

## Model Design of an Internet-Based Epidemiological Survey Data Collection Custom System

### Design of System Data Acquisition Based on Internet

**Figure 3:**
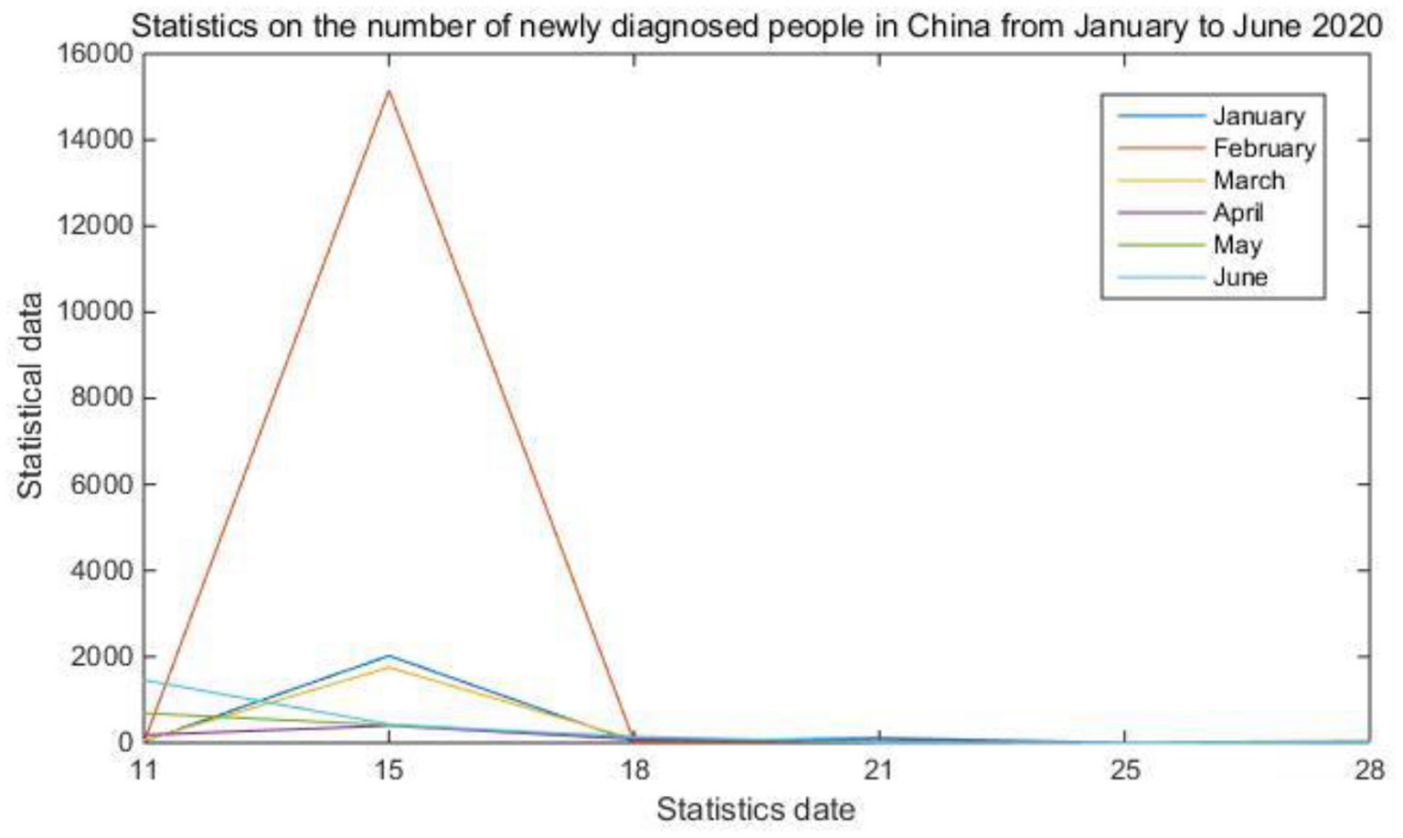
Statistics of newly diagnosed people in China.

In the process of epidemiological investigation, the most important link is the collection of data on the population infected by the epidemic every week, every day or even every hour. The more infectious and harmful viruses, the more necessary to update the system in time. The data allows officials and the masses to accurately grasp the changing status of communication. The absolute amount of intelligence of a data only reflects the scale of the current period. It is difficult to tell whether it is large or small in isolation. Only by comparing the data in different periods and observing its trajectory can the cause of the change be effectively analyzed and the correct judgment can be made. Incremental analysis is mainly to calculate the growth rate. Through the year-on-year growth rate, the chain growth rate and the statistical graph, the system can better show the changes in the epidemic growth rate. Table 1 and Figure 3 show statistics on the number of newly confirmed cases of the epidemic in China from January to June 2020.

**Table 1:** Statistics of newly diagnosed people in China.

	**11**	**15**	**18**	**21**	**25**	**28**
January	34	4	59	182	688	1,459
February	2,022	15,152	1,751	399	411	430
March	25	29	84	82	144	128
April	113	52	21	37	14	22
May	1	9	6	13	7	0
June	7	45	37	21	29	14

The display of collected data is the most important function in the epidemic disease customization system. It can be seen from Table 1 and Figure 3 that although the COVID-19 outbreak had cases as early as December 2019, the number of confirmed cases did not increase significantly until late January 2020. February became the peak of the outbreak, with the highest number of confirmed cases in a single day reaching 15,152. Compared with the data indicators in the table, the statistical graph can clearly reflect the changes in the growth rate and speed more intuitively.

**Figure 4:**
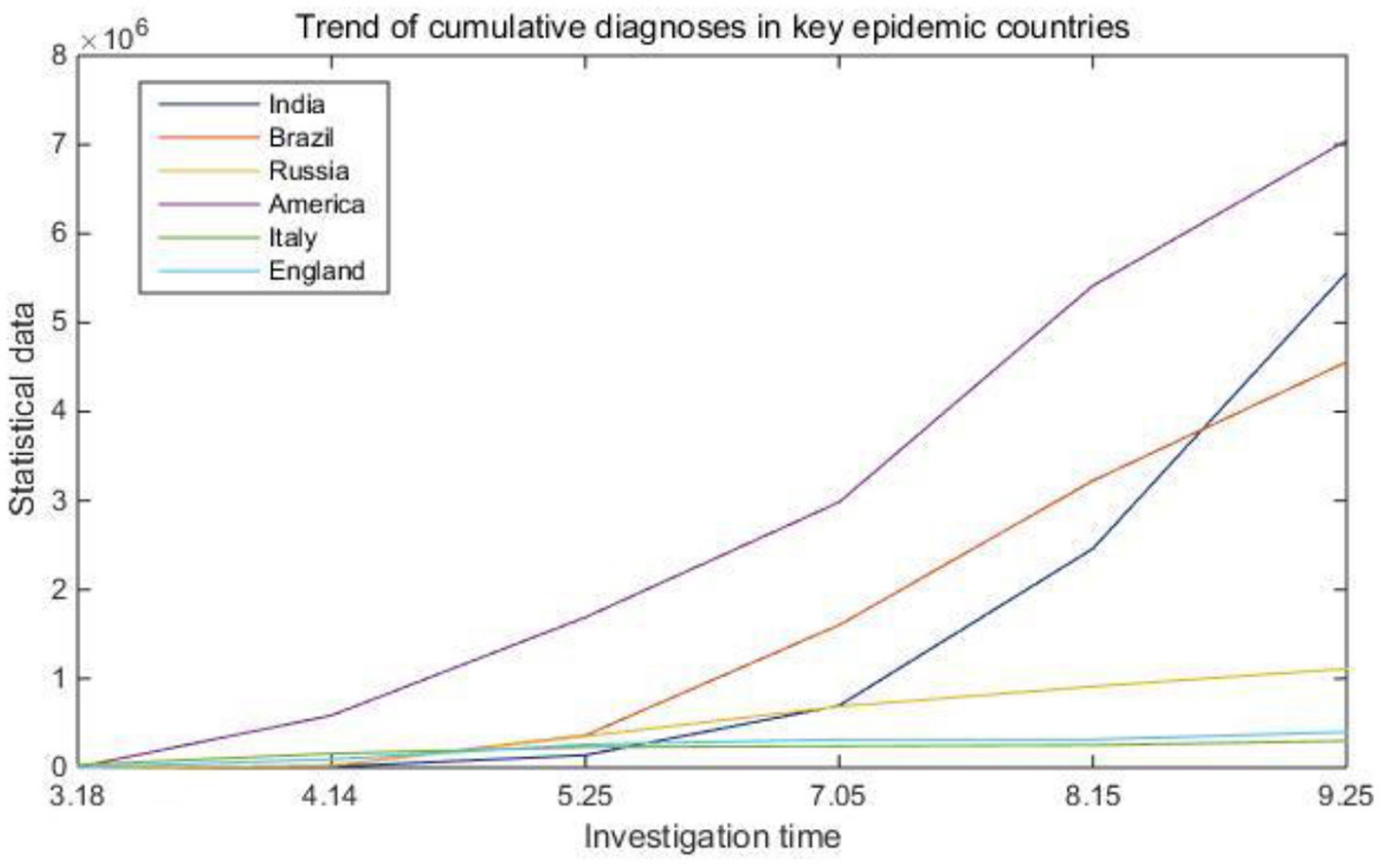
Trend of cumulative diagnoses in key epidemic countries.

The proportional analysis method is the main method to analyze qualitative data. A single analysis of the absolute quantity of the frequency of a certain data item in qualitative data does not make much sense. Only by analyzing the proportion of each data item in the total and the proportional relationship between them can we get the results that people care about. The proportional analysis method is to first calculate the percentage of each option frequency of the indicator to the total frequency to obtain the current period, and then further use the incremental analysis method to study the changing law of the data in each period. In the epidemiological survey data collection system, in order to grasp the changes in the number of confirmed cases of the global epidemic and promote the development of information globalization, this article adds the data changes of the world epidemic to the system. Table 2 and Figure 4 show the cumulative confirmed trends in key epidemic countries.

**Table 2:** Trend of cumulative diagnoses in key epidemic countries.

	**3.18**	**4.14**	**5.25**	**7.05**	**8.15**	**9.25**
India	151	10,541	141,228	697,887	2,461,190	5,562,663
Brazil	291	23,955	363,211	1,603,055	3,224,876	4,558,040
Russia	147	21,102	353,427	686,777	912,823	1,111,157
America	6,552	587,752	1,689,727	2,983,155	5,415,666	7,046,216
Italy	31,506	159,516	229,858	241,611	252,235	299,506
England	2,626	94,823	260,916	314,992	315,600	401,127

From Table 2 and Figure 4, we can clearly see the changes in the number of confirmed cases in several key epidemic countries such as the United States, India, and Brazil. As early as March, the World Health Organization defined the COVID-19 outbreak as a global pandemic. Most epidemics will only spread in local areas. If there is a pandemic on a global scale, relevant organizations and the masses need to pay more attention to it. Considering this kind of situation, this article carried out business reorganization by extracting the commonality of business process modeling when designing the system data collection. In this way, a data collection system with a universal scope can be better designed to collect and summarize epidemic data information on a global scale.

### Data Analysis of Epidemiological System Based on Internet

The most important feature of a pandemic virus is that it can spread widely in a short period of time. Therefore, the epidemiological data system has a very important goal, which is to conduct disease transmission risk assessment by collecting data and information, and to give professional prevention suggestions. During this COVID-19 epidemic, officials will report the changes in the number of infections in various provinces and cities every day, and some service systems will even provide the location of the patient's residence to increase the vigilance of nearby people.

**Figure 5:**
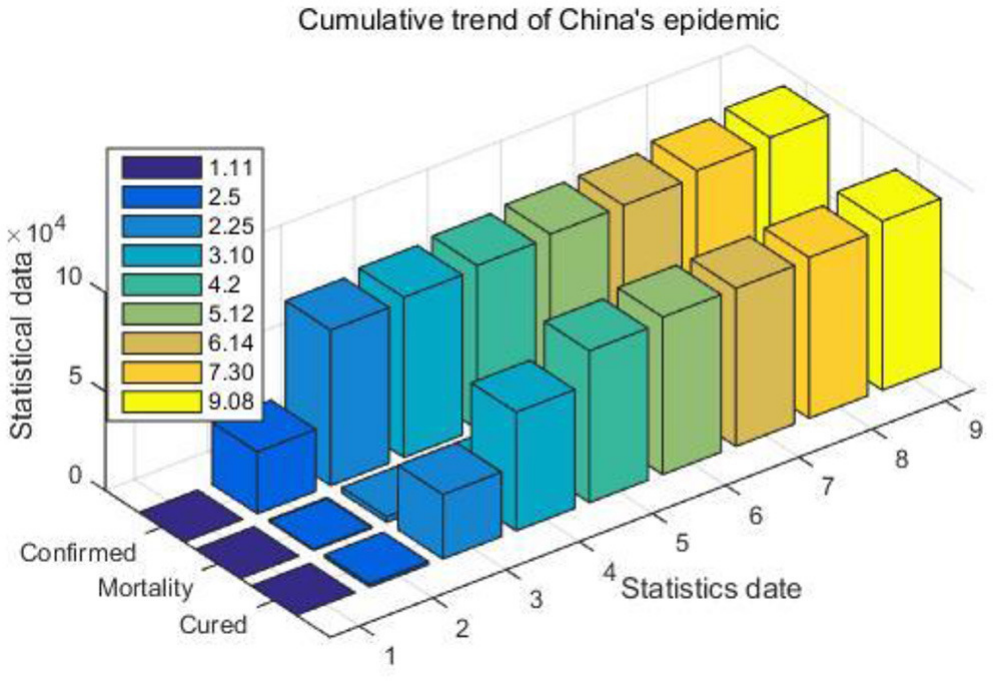
Cumulative trend of China's epidemic.

In the design concept of this article, an excellent epidemiological data collection system can become a platform for communication between officials and the masses, allowing the masses to learn about the changes in the epidemic in real time. For these considerations, this article believes that data mining algorithms should be introduced into the data collection system, that is, to select valuable data from massive amounts of big data, and coordinate the data for different purposes, and present the summary results clearly and easily to the system user. Table 3 and Figure 5 show the cumulative trend of the epidemic in China.

**Table 3:** Cumulative trend of China's epidemic.

	**1.11**	**2.5**	**2.25**	**3.10**	**4.2**	**5.12**	**6.14**	**7.30**	**9.08**
Confirmed	41	31,211	78,630	80,924	82,802	84,458	84,778	88,122	90,582
Mortality	1	637	2,747	3,140	3,331	4,644	4,645	4,668	4,740
Cured	6	1,542	32,531	59,982	76,785	79,594	79,913	81,227	85,411

It is obvious from Table 3 and Figure 5 that from January to March, the number of confirmed cases of COVID-19 was in an explosive growth period in my country. During this period, the system will update the data accumulation situation in real time at a frequency of half an hour, and publish the latest epidemic information of various provinces and cities in the system news template.

Compared with some data changes with obvious regularities, the survey data of epidemics often have more uncertainties. When an acute respiratory infectious disease like COVID-19 appears in its early stages, no one would have expected that it will eventually develop into a global pandemic that infects tens of millions of people. However, as time goes by, it is possible to predict future trends from gradually stable data changes. To infer future trends, regression analysis methods are usually used. Regression analysis is a statistical method and technique that examines the quantitative changes between a number of independent variables x and dependent variables y. Regression analysis hopes to obtain a mathematical expression of the connection between the independent variable and the dependent variable, and estimate and predict the dependent variable through the known independent variable. The general steps of regression analysis are as follows: starting from the sample data, determine the mathematical relationship between the variables; estimate the regression model parameters; statistically test the relationship to find out the significant variables.

**Figure 6:**
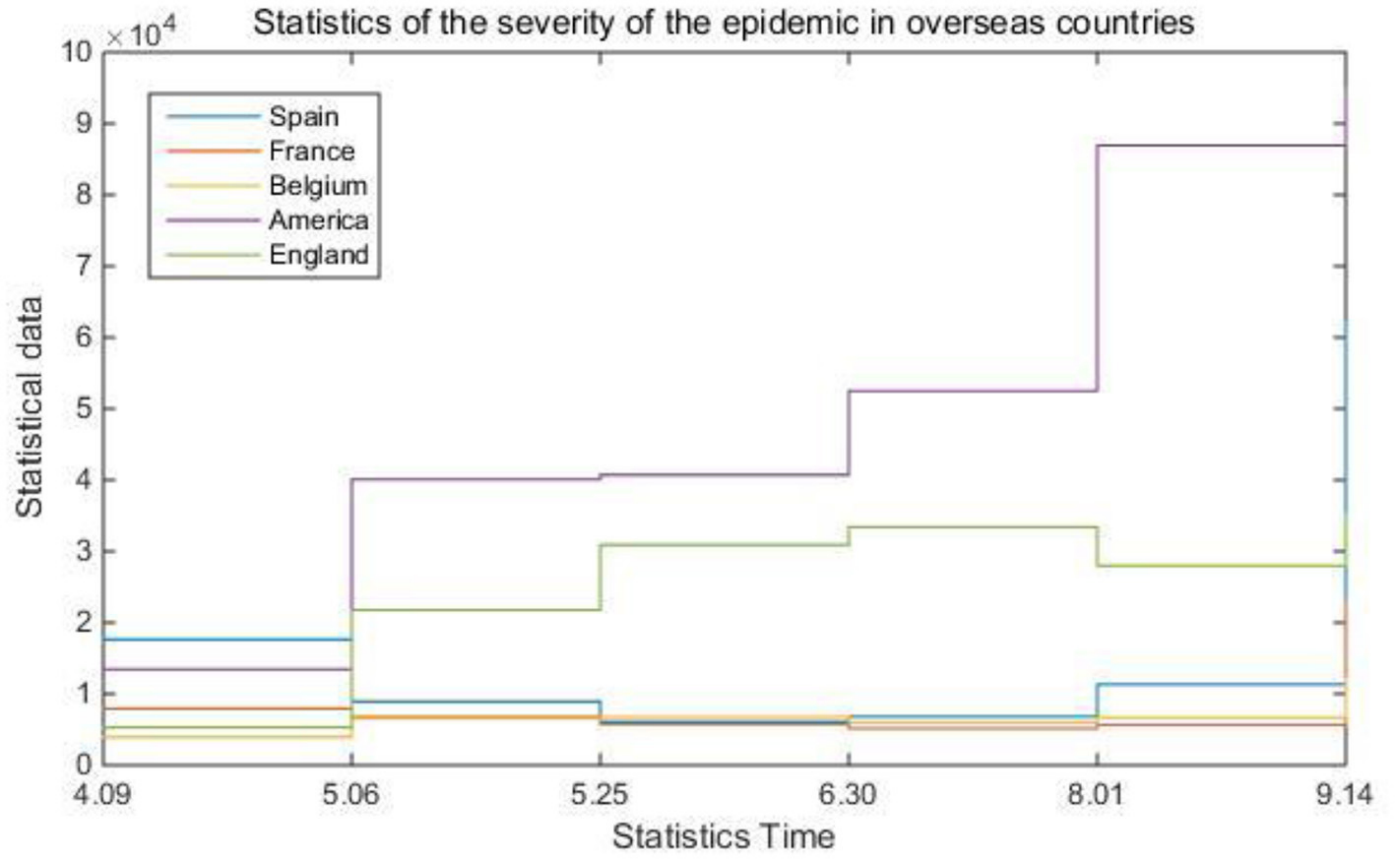
Statistics of the severity of the epidemic in foreign countries.

In real life, there are many uncertain factors that affect the spread of epidemics. In this case, computer technology is needed as assistance, such as eliminating useless information that interferes with prediction through data mining algorithms, and improving system information processing through backpropagation algorithms ability. Table 4 and Figure 6 are statistics on the severity of the epidemic situation in overseas countries.

**Table 4:** Statistics of the severity of the epidemic in foreign countries.

	**4.09**	**5.06**	**5.25**	**6.30**	**8.01**	**9.14**
Spain	17,644	8,930	6,071	6,814	11,324	62,227
France	7,936	6,752	5,778	5,170	5,692	22,723
Belgium	3,935	6,565	6,782	6,019	6,697	12,109
America	13,476	40,086	40,708	52,496	86,940	94,957
England	5,280	21,778	30,905	33,439	27,993	35,183

**Figure 7:**
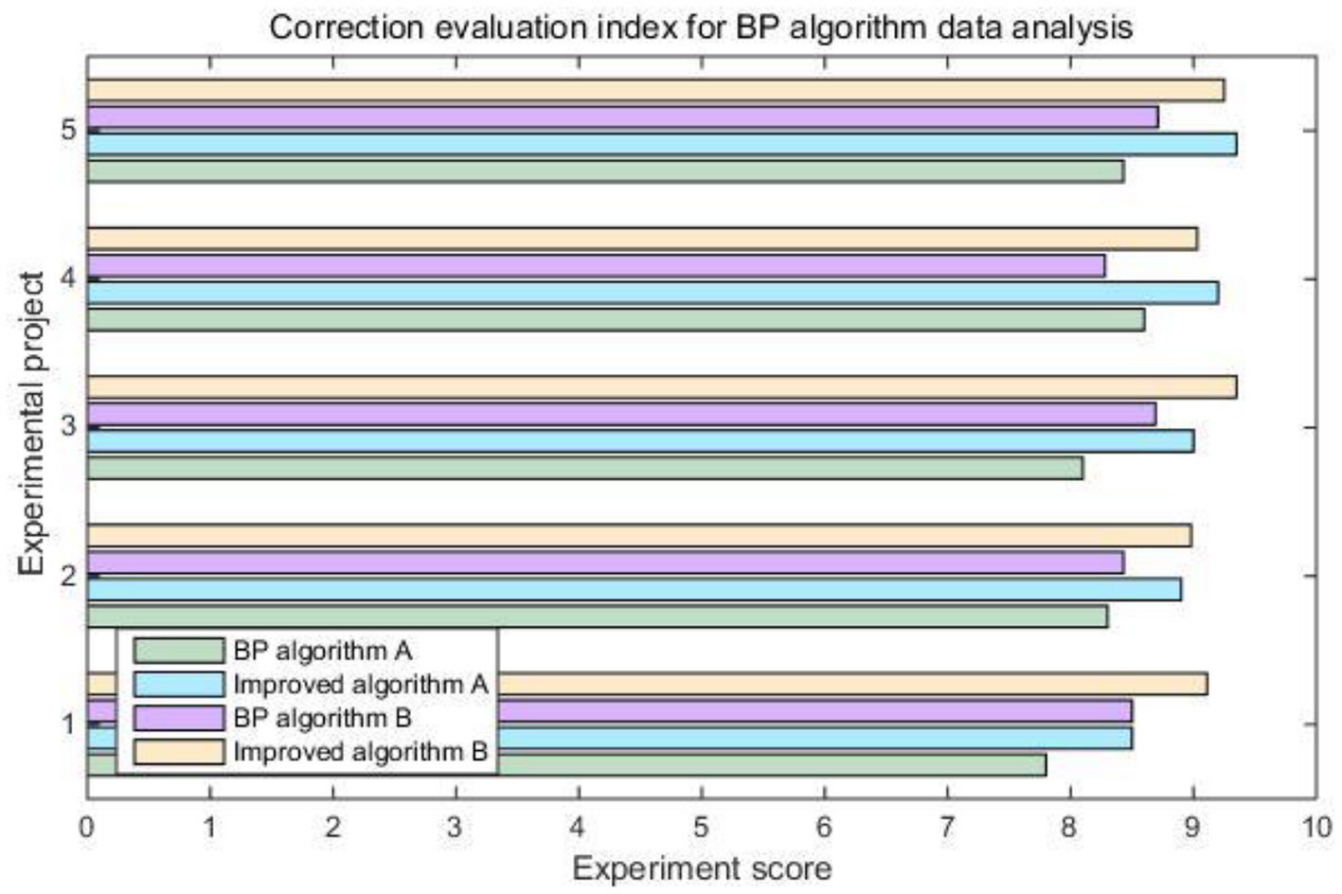
Correction evaluation index for Bp algorithm data analysis.

Combined with the system's built-in computer intelligent algorithm, this article gives the concept of the epidemic severity index. That is to say, comprehensively refer to multiple data such as the global population, the global urban construction area, the number of existing confirmed populations, and the existing confirmed population density to judge the current severity of the epidemic in different countries. It can be seen from Table 4 and Figure 6 that the severity of the epidemic situation in most countries has fluctuated to varying degrees. In contrast, the severity of the epidemic index in the United States has basically remained rising. Figure 7 shows the correction evaluation index of the data analysis in the simulation experiment of the backpropagation algorithm after the weight is corrected by the gradient method.

According to Figure 7, it is not difficult to see that the back propagation algorithm after modifying the weight by gradient method can play a better information processing effect in the epidemiological survey data collection system. The optimized correction evaluation index has been increased from 8.384 to 9.067. When combined with data mining algorithms, it can effectively improve the efficiency of data processing.

### TInfluence of Internet-Based Epidemiological System on Information Management

**Figure 8:**
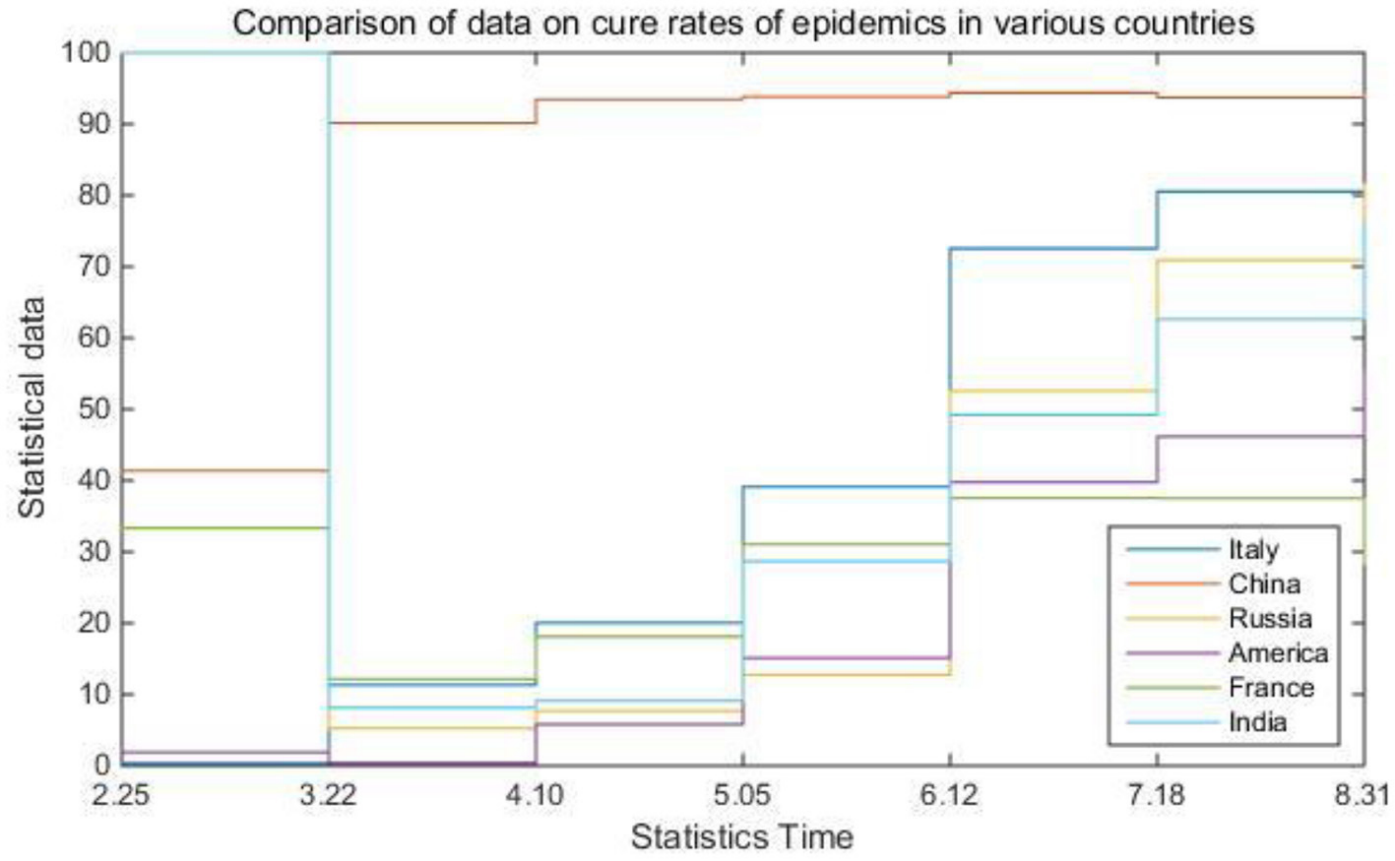
Comparison of data on cure rates of epidemics in various countries.

In practical problems, the factors that affect the dependent variable are very diverse. This regression of one dependent variable with multiple independent variables is called multiple regression. In real life, the impact of the COVID-19 epidemic on all parts of the world is huge. It not only threatens human life, health and safety, but also affects the development of the global economy. In addition, the epidemic has also had a considerable impact on the original global information management system and digital business in life, making people aware of the importance of information and data management in the Internet era. Table 5 and Figure 8 compare the data on the cure rate of epidemics in various countries.

**Table 5:** Comparison of data on cure rates of epidemics in various countries.

	**2.25**	**3.22**	**4.10**	**5.05**	**6.12**	**7.18**	**8.31**
Italy	0.35%	11.33%	20.05%	39.11%	72.56%	80.54%	77.75%
China	41.37%	90.16%	93.48%	93.84%	94.37%	93.71%	94.09%
Russia	100%	5.23%	7.69%	12.79%	52.57%	70.93%	81.52%
America	1.89%	0.38%	5.82%	15.12%	39.77%	46.16%	55.49%
France	33.33%	12.17%	18.13%	31.04%	37.54%	37.5%	28.36%
India	100%	8.13%	9.12%	28.69%	49.21%	62.61%	76.63%

It can be seen from Table 5 and Figure 8 that during the epidemic, China's cure rate has a very obvious advantage compared with other countries. This is naturally due to the country's effective epidemic prevention measures and treatment methods, but on the other hand, Official and reasonable information management methods have also played a good role in it. During the epidemic, the state and government update official survey data in real time through open channels, and the public can learn about the severity of the local epidemic and various epidemic prevention policies through the Internet. In this information age, if there is no official channel to disclose professional data in a timely manner, the masses are easily affected by the wrong information spread by some criminals. From this perspective, it is enough to prove how important a professional information disclosure platform is. The Internet-based epidemiological survey customized system model established in this article is to integrate various epidemiological data so that people can correctly understand the spread of epidemics and promote the development of preventive medicine.

## Conclusions

Traditional epidemiological surveys mostly fill out paper questionnaires in person or over the phone. However, with the development of the Internet, this type of survey and data methods have obviously no longer adapted to the pace of the times. The survey data involved in epidemiology not only has many types, but also has higher requirements for real-time performance. Therefore, this article believes that the establishment of a special epidemiological survey data system through the Internet can better improve work efficiency and achieve scientific realization Information management. This article uses COVID-19 as a typical case, analyzes the possible problems and obstacles in the data collection process, and develops a customized epidemiological data collection system model with sufficient professionalism and practicability.

This study analyzed the business requirements of the epidemiological survey system, and designed a database table combining the attribute data of the survey unit and the spatial data. Based on the global COVID-19 epidemic survey data, this article analyzes the number of confirmed cases, severity index, cure rate and other indicators in several major epidemic countries from the perspectives of total indicators, incremental analysis methods, and proportional analysis methods, combined with tables in the system Data and statistical charts can clearly grasp the spread of epidemics. During this epidemic, my country's excellent information management strategy reflected to a certain extent the importance of data disclosure in the information age. Establishing an Internet-based customized epidemiological survey system model can not only integrate various epidemiological data, but more importantly, allow people to correctly understand the spread of epidemics and promote the development of preventive medicine.

In the research process, this article has achieved certain results. Nevertheless, due to various constraints, the research has certain limitations. In future research, this article believes that the experimental results can be further improved from the following perspectives: (1) Improve data mining algorithms and continuously improve the system's ability to analyze data in the era of big data; (2) Optimize the data management system The query function provides users with richer query services; (3) Improve system security protection capabilities to ensure the accuracy of data transmission.

## Data Availability Statement

The original contributions presented in the study are included in the article/supplementary material, further inquiries can be directed to the corresponding author/s.

## Author Contributions

All authors listed have made a substantial, direct and intellectual contribution to the work, and approved it for publication.

## Funding

This work was supported by the National Natural Science Foundation of China (Grant No. 61704138) and the Natural Science Foundation of Shaanxi Province (Grant No. 2020JQ-655).

## Conflict of Interest

The author declares that the research was conducted in the absence of any commercial or financial relationships that could be construed as a potential conflict of interest.

## Publisher's Note

All claims expressed in this article are solely those of the authors and do not necessarily represent those of their affiliated organizations, or those of the publisher, the editors and the reviewers. Any product that may be evaluated in this article, or claim that may be made by its manufacturer, is not guaranteed or endorsed by the publisher.
